# Patterns of University Students’ Risky Sexual Experiences and Their Characteristics

**DOI:** 10.3390/ijerph192114239

**Published:** 2022-10-31

**Authors:** Maria Łukaszek

**Affiliations:** Department of Resocialization Pedagogy, Faculty of Pedagogy, University of Rzeszów, 35-310 Rzeszów, Poland; mlukaszek@ur.edu.pl

**Keywords:** risky sexual behaviours, students’ sexual behaviours, students’ sexual health, determinants of sexual behaviours, sex after alcohol, sexual violence, providing paid sexual services, Poland

## Abstract

In Poland, there is little research on university students’ risky sexual behaviours. Additionally, existing studies analyse the behaviours selectively and do not group them into clusters. Hence, effective prevention is impossible. The research aims to gather information regarding the prevalence, forms and clusters of students’ risky sexual experiences. In 2019, a cross-sectional study was conducted in 12 universities in south-eastern Poland on a random sample (*n* = 2764). Fifteen risky sexual experiences, both condom-protected and unprotected, were analysed; they were mainly unprotected vaginal, oral, anal contacts; protected and unprotected sexual experiences while intoxicated and with unknown persons. Thanks Ward’s hierarchical method, four inseparable clusters of students with similar risky experiences were distinguished. Their dominant features were: (A) (24% of interviewees)—drunk partners, (B) (4.8%)—partners intoxicated with drugs, (C) (3.1%)—partner abuse and exceeding partner sexual norms, (D) (17.8%)—the anonymity of partners and going beyond the convention. It was stated that 60.3% of the respondents do not belong to any of the identified clusters. Sex education and the promotion of student sexual health should be intensified; the development of attitudes of avoiding risky sexual behaviours and dealing with their consequences should be considered.

## 1. Introduction

When addressing the subject of risky sexual experiences, it should be emphasized that their consequences relate to many spheres of human functioning. They can be considered in the individual (relating to the sexual, reproductive, mental health of an individual) and collective dimension, such as the social and economic costs of maintaining public health [1]. Risky sexual behaviours in students should be additionally analysed from the perspective of social influence. Students in their local communities, especially in Poland, have a high social status and authority [2,3,4,5], which means that they are perceived as significant people. Their behaviours and beliefs can, therefore, constitute a desired and replicated model of behaviour.

Machaj et al., state that any sexual behaviour that: (1) leads to the loss of health (permanent or temporary), body injury (permanent or temporary) or poses a threat (direct or indirect) to life; (2) evokes difficult emotions, unfavourable mental states considering consequences, (3) risks pregnancy, (4) leads to a lack of control over one’s own body and/or mind, e.g., while intoxicated with psychoactive substances, but also (5) a sexual behaviour with a non-sexual goal, (6) a behaviour significantly exceeding human values, and (7) a behaviour which violates the norms of social coexistence should be considered as risky, with particular emphasis on the possibility of contracting sexually transmitted diseases [6]. According to French, risky sexual behaviours should be considered both voluntary contacts and the ones to which one was forced [7].

In the studies that have carried out worldwide, the risks connected to sexual behaviours were mainly considered from the perspective of sexually transmitted infections, as presented by national centres for disease control and prevention (e.g., the National Institute of Public Health, National Institute of Hygiene (PZH)in Poland [8,9], Centers/ for Disease Prevention and Control (CDC) in the USA [10]). They have also been linked to the problem of unwanted pregnancies and dangerous abortions [10,11]. The problem that is increasingly being stressed, especially by WHO [11,12], is sexual violence.

The above-mentioned areas of interest were not analysed without reason. It is estimated that young people at the age of 15–24 acquire almost half of all new sexually transmitted diseases [13,14]. In Poland, in 2009–2020, people aged 20–29 accounted for 32% of newly detected HIV infections [8,15]. According to WHO reports, 1 in 5 women and 1 in 13 men declare that they were sexually abused as children [12]. In the USA, about 8% of girls and 0.7% of boys under 18 have experienced rape or an attempt of rape [16]. In Poland in 2011, 6% of men and 20% of women admitted to having experienced sexual violence [17].

The findings set the direction for research carried out in groups of adolescents around the world, including students. The largest part of studies related to risky sexual behaviours in this group concerned the following issues (most often considered together): early sexual initiation, frequency of vaginal, oral and anal unprotected intercourse, number of sexual partners, sexual contacts with random partners and the use of contraception [18,19,20,21,22,23,24,25,26,27,28]. The basic conclusion drawn from the research is that students are a group who is particularly prone to engaging in risky sexual behaviours. Moreover, Caldeira’s team, who carried out longitudinal research, proved that during the course of studies, the indicator of various risky behaviours in the research sample increased by 40% or even by 70% [29].

Most studies have attempted to isolate risk factors for engaging in particular behaviours. They were usually individual determinants: related to the characteristics of the respondents (e.g., gender, age, sexual orientation) or experiences of other risky behaviours (e.g., getting drunk, using drugs, participating in fights, participating in discos [26,29]. The social context, including family and peers, was taken into account much less often [22,24,26,27]. Some studies also concerned the relation between childhood abuse [30] and attachment style [31] in terms of risky behaviours.

A separate part of the studies carried out in groups of students concerned engaging in commercial sexual activity, which shows that 5–17% of them provided various sexual services, both in the form of sexual contacts and erotic dance, stripteases or sharing their erotic photos [32,33,34,35,36].

At present, more and more research is being related to the problem of sexual violence, especially that experienced by students during their university education [37,38].

Despite the fact that much of the research on the risky sexual behaviour of students have been carried out worldwide to date, due to the small sample sizes and methodological differences (e.g., online or face-to-face research), it is very difficult to draw unambiguous conclusions [26,28,39].

To date, little research on students’ sexuality has been carried out in Poland, and particularly on their engaging in various risky sexual behaviours. Most of the studies were performed on small samples, in single research centres (usually the largest ones in the country or region). Students were often limited to selected fields of study (e.g., medical students). Therefore, the existing reports do not provide a complete and detailed picture of the risky sexual experiences of all students. For this reason, it is worth recalling conclusions from not only research conducted among students, but also from representative nationwide samples.

Over the last twenty years, there have been huge changes in sexual behaviours in Poland, especially among adolescents and so-called young adults. A significant reduction in the age of the first sexual initiation was found, especially in the group of women (in 2001–2017, for men from 18.38 to 18.28, for women from 19.19 to 18.45 [40]).

There has been a significant increase in the number of sexual partners, especially among women. From 1997 to 2017, the average number in the group of men increased from 4.5 to 5 and in the group of women from 2 to 3.8 [40]. The increase concerns mainly adolescent groups. As far as students aged 18–19 years old are concerned, intercourse with five or more partners was declared by 23% of men and 6% of women, and in the group of students aged 19–20by 23% of men and 11% of women [18]. The average number of students’ sexual partners is 3.4 [19].

Speaking of sexual habits of Poles, especially that of young people, it should be underlined that forms of sexual activity that were previously rare have become more common. In the years 1997–2017, there was a significant increase in the percentage of people with experience of oral contacts (five times among women and almost four times among men) and anal contacts (almost three times among women and two times among men) [17,40,41]. In 1997–2005, the percentage of Poles having experienced collective sexual contact increased four times [17,41]. In the years 1997–2011, there was a threefold increase in the percentage of persons who used sexual services [17,40,42]. In 1997–2005, the percentage of men declaring the provision of sexual services increased fivefold and the percentage of women fourfold [17,41]. From 2005 to 2011, the percentage of people who declared having been forced into unwanted sexual intercourse increased many times (six times among men and seven times among women [17]).

The nationwide (online) study of students reveals that 78% of the respondents are sexually active, 19% have experiences of casual sex, and 27% had intercourse while intoxicated with alcohol. The groups most exposed to risky sexual behaviours are mainly: homosexual men, bisexual women, art and military students, and people who drink alcohol [22].

Despite intensive changes in the sexual habits of Poles, the research prepared for the National AIDS Center shows that in 2015, in the group of sexually active Poles, 52% never used a condom and only 18% did so regularly [43]. A nationwide study from 2017 proved that, in the year preceding the study, 39.9% of people never used a condom during vaginal contacts, 77.1% during oral contacts, and 62.5% during anal contacts [17]. As far as students are concerned, 60% confirmed consistent use of condoms during vaginal intercourse and 5.2% during anal intercourse [19].

For the research area, the Podkarpackie region was chosen. It is located in south-eastern Poland and borders Ukraine and Slovakia. It is commonly regarded as the mainstay of traditional values, including family and religious ones. The beliefs may be reinforced by facts, e.g., the Podkarpackie Voivodeship ranks second in the country in terms of inhabitants’ declarations of satisfaction with their marriage or partnership relationship [44] and the dioceses of Rzeszów and Przemyśl are famous for the zealous participation of the faithful in Sunday services [45].

The stereotypical image, however, has undergone significant changes, which certainly had an impact on the sexuality of its inhabitants, especially the young generation, including students. It should be noted that, in recent years, there has been a sharp increase in the mobility of the Podkarpacie region residents in the areas of education, work and tourism [46]. The rate of temporary emigration, both foreign and domestic, has also significantly increased [47]. Moreover, there is a growing divorce rate (by 23% when comparing 2019 to 2010) [48]). The percentage of people intoxicated with psychoactive substances (especially alcohol and marijuana consumers) is growing rapidly [49]. In recent years, a weaker commitment to religious practices has been also observed (e.g., the participation rate of Catholics from the Rzeszów and Przemyśl dioceses in messes in 2019 was 10% lower than in the years 1992–2003) [45,50].

The research subjects are students, as this is a group of young adults that, in Poland, is generally not included in social prevention, especially in the context of risky sexual behaviours (cf. [51]). This probably results from the misconception that students have a high level of knowledge of human sexuality [52] (which allegedly translates into their understanding and ability to avoid risky situations). Other competences of students (e.g., ease of establishing interpersonal relations, both direct and via instant messaging, free use of foreign languages, mobility) and their habits (e.g., related to spending free time and the use of psychoactive substances in a group) are ignored, although they may be potential factors which generate risky sexual situations.

The subject of the present research is students’ risky sexual experiences, which are analysed by the author, considering a wide range of adverse effects [6]. The consequences are not only sexually transmitted infections and unplanned and unwanted pregnancies. Traumatic experiences and physical injuries resulting from the experience of physical, mental or sexual abuse are also crucial. Not only do they cause physical suffering, but they also disturb the sense of security and the ability to feel adequate self-esteem as a sexual partner, which results in significant loss. They also evoke feelings of disappointment and regret, due to, e.g., accidental intercourse with an unknown person [53], establishing an intimate relationship with someone whom a person normally does not desire [54], a belief that the decision about intercourse resulted only from the fact of being intoxicated [55], and also because of the failure to use a condom and potential harm this entails [56]. It must not be forgotten that one of the unpleasant consequences of risky sexual experiences, especially contacts with unknown partners, is a reduced chance of their co-responsibility for the undesirable effects of the activity (e.g., HIV infection, pregnancy). The key and, additionally, the permanent effect of risky sexual experiences is the fact that, when repeated many times, they result in disturbed sexual scripts (fixed activity patterns in the relationships of intimate partners, but also preferences as to the behaviour of partners and the acceptance of specific actions in the sexual sphere [57,58]).

The research aim is to gather information regarding the prevalence, forms and clusters of university students’ risky sexual experiences. The author attempted to answer the following questions:

- What is the prevalence and forms of students’ risky sexual experiences?
- Do students form clusters that are similar to each other in terms of risky sexual experiences, and if so, what are they like?
- Which of the examined factors enumerated below characterise and mark out the respondents with particularly risky sexual experiences in terms of the defined clusters of risky sexual behaviours?
  ° Socio-demographic (gender, age, place of growing up, student’s parents’ relationship model, sexual orientation, religious commitment).
  ° Self-independence dimensions (period of living outside the family home, student’s support sources during studies, being in a marriage or stable partnership).
  ° Way of fulfilling the role of a student (field of science studied, grade average).
  ° Participation in social meetings while intoxicated with psychoactive substances (alcohol, drugs).

## 2. Materials and Methods

### 2.1. Study Design and Sample

The material presented in the article comes from the cross-sectional study entitled ‘Attitudes of students of the Podkarpackie universities towards HIV/AIDS’, implemented by the author in 2019 in all 12 universities in the Podkarpacie region.

The research sample consisted of 2764 students (59.7% were women and 40.3% men), who constituted 5.9% of all people studying in the Podkarpackie voivodeship in the current academic year. The research participants were only Poles (there were several non-Polish students who were included in the random sample but, due to poor knowledge of the Polish language, they refused to participate in the study).The average age of the respondents was 22.9 years. The sample was selected randomly; however, it was selected from among men and women studying in particular fields of science. The procedure reflected the structure of the total number of students in the Podkarpacie region in terms of gender and the field of science studied (based on the data of the Central Statistical Office [59]; more details can be found in the preliminary research report, which was published on the website of the National AIDS Center [52]). The characteristics of the sample regarding the independent variables analysed in the present material are included in Table 1.

### 2.2. Survey Measures, Procedure

The research tool (a survey questionnaire) used in the study consisted of 44 questions or blocks of questions. The presented material contains the results of 13 questions on independent variables and one block of questions on dependent variables.

Risky sexual experiences were measured using a block of 15 questions about sexual behaviours, each of which addressed two situations: with or without the use of a condom (Table A1). The respondents were given one instruction for the entire block: “Indicate what experiences you have had in your life. Refer to all experiences by putting a cross in the appropriate column”. In the survey, below the instruction, there was a table with a set of 15 behaviours (divided into protected and unprotected contacts—30 lines in total):

1. Sexual contact with a completely unknown person;
2. Sexual contact with a poorly known person;
3. Oral contact with ejaculation into the mouth;
4. Vaginal sex;
5. Anal sex;
6. Sharing sex toys (e.g., vibrators, balls);
7. Using sexual services (oral, vaginal or anal contact) for money or other favours in exchange;
8. Providing sexual services (oral, vaginal or anal contact) for money or favours in exchange;
9. Group sex (with several people at once or alternately);
10. Coercing somebody to sexual contact (oral with ejaculation in the mouth, vaginal or anal);
11. Being coerced to sexual contact (oral with ejaculation in the mouth, vaginal or anal)
12. Sexual contact when only you were under the influence of alcohol;
13. Sexual contact when both you and your partner were under the influence of alcohol
14. Sexual contact when onlyyou were intoxicated with drugs;
15. Sexual contact when both you and your partner were intoxicated with drugs.

In total, the respondents gave 30 answers on a three-point scale: the behaviour which never occurred, it occurred once, or it occurred more than once. The interviewees had a right not to answer all questions, which was marked as no answer in the summary tables.

There were thirteen questions on independent variables (Table 1) concerning the following: gender, age, place of growing up, parents’ relationship model, sexual orientation, religious commitment, period of living outside the family home, student’s support sources during the studies, being in a stable relationship or marriage, a field of study, grade average from the previous term, participation in social meetings while intoxicated with drugs and participation in social meetings under the influence of alcohol (categories of variables are included in the Appendix A). To ensure respondents could properly understand the questions related to the state of ‘alcohol intoxication’, the questionnaire included its definition and a list of its typical indicators such as, e.g., coordination disorders occurring after alcohol consumption (swaying, falling over), speech disorders (slurred speech), decreased intellectual performance (errors in logical reasoning, lack of criticism), inadequate emotional reactions, significantly reduced ability to control one’s behaviour, increased sleepiness.

The present research was carried out in 12 universities in direct contact with the respondents. The study was preceded by a pilot study conducted among students of the University of Rzeszów. During the research implementation, the respondents received comprehensive information on the goal and the subject of the research, on their complete anonymity and voluntary participation in the survey, the method of analysing the collected diagnostic material and spreading the results, and the possibility of obtaining advice on the issues referred to in the survey. The final consent for the research was expressed by the students after their discussion with the interviewers and reviewing the questionnaire. After completing the questionnaires, the students put them in envelopes and returned them sealed. Then, the interviewing team provided the respondents with educational materials on HIV/AIDS, which included contact details of institutions providing advice and support in the field of the survey. The interviewees were also provided with the possibility of an individual conversation on the topics included in the survey with a sex educator.

### 2.3. Research Ethics

The research fully considered the recommendations contained in the Helsinki Declaration. The research was approved by the Bioethics Committee of the Regional Medical Council in Rzeszów (Resolution No. 40/B/2019 of 11 April 2019).

### 2.4. Analytical Approach

In the first stage of the research, 15 risky sexual experiences were analysed (only descriptive statistics were presented). They were divided according to two situations (with and without a condom). Total percentages (for the two situations) related to undertaking the enumerated forms of activity were also distinguished. This allowed for us to check the percentage of people, among those who had risky experiences, who did not use condoms. The comparisons (within individual sexual behaviours) were also made, and on this basis, the ratio of repeated experiences to single ones was established.

In the second stage, in order to distinguish clusters of students with similar risky sexual experiences, the results were analysed using the Ward’s method, which is one of the hierarchical agglomeration methods. This allows for us to estimate the distance between clusters using analysis of variance. Twenty-six behaviours considered to be risky (vaginal, oral, anal contacts and the use of sex toys with a condom were considered as safe, so they were not taken into consideration in the analysis of risky sexual experiences clusters (Table A1)) were assigned two risk levels: low, where risky behaviours were incidental, and high, where the behaviours were repeated. The identified clusters are inseparable, which means that a person from one cluster can also belong to other clusters.

In the third stage of the analysis, the author presented the characteristics of people who have similar risky sexual experiences in terms of the isolated clusters, including 13 factors, which form the four groups (socio-demographic and self-independence ones, the way of fulfilling the role of a student, participating in social meetings/parties when intoxicated with psychoactive substances).

The analyses were performed with the use of the specialized STATISTICA 12 statistical package (StatSoft, Cracow, Poland).

## 3. Results

### 3.1. Risky Behaviours—Prevalence and Forms

In the first stage of the analysis, an attempt to determine the prevalence and forms of students’ risky sexual experiences (vaginal, oral and anal contacts and the use of sex toys while using a condom were considered as safe) was made. The author also tried to determine the percentage of people, in the group of all who had particular risky experiences, did not use condoms. The author also aimed to compare repeated experiences in relation to single ones (within the 15 sexual behaviours).

The research results (Table 2) show that a high percentage of students engage in various risky sexual behaviours. The most frequent ones are unprotected vaginal contacts (39.7%), oral contacts with ejaculation into the mouth (21.7%) and anal contacts (13.6%). Almost one-third of respondents (28.3%) stated that they had intercourse with their partners intoxicated with alcohol while they were also under the influence of alcohol. Additionally, 71.7% of them had such experiences without using condoms. A high percentage of the respondents (20.9%) also reported experiences of sexual contacts in a state of alcohol intoxication. It should be underlined that 79.6% of them established such relationships without condom protection. During the analyses, it was found that students had numerous sexual contacts with completely unknown (13.6%) or very little-known persons (16.5%). Despite the high risk, about 60% of the respondents did not always use a condom on these occasions.

It should be noted that a significant percentage of the respondents admitted to high-risk sexual experiences, i.e., engaging in intercourse while intoxicated with drugs (5.4%) or when both they and their partners were so (5.3%), providing (2.1%) and using sexual services (3.2%) and group sex (2.8%). Not only do they carry a risk to sexual and reproductive health, but also pose threat to personal safety, due to the high probability of sexual and physical aggression. The results show that 2.6% of the respondents declared that they had used sexual violence against others, and 3.7% claimed to have been a victim of sexual assault in the past.

The collected material proves that the majority of the surveyed students who engage in various sexual behaviours are not consistent in the use of condoms. As far as all 15 sexual experiences are concerned, 60–87% of the respondents declared condom-unprotected sexual contacts.

When it comes to creating sexual scripts, it is also important to underline that the experiences of risky sexual contacts declared by the respondents are most often repeated many times. Considering all risky sexual behaviours, it was found that the students reported repeated risky behaviours at least twice as often as single events. The greatest predominance of repeated behaviours over single ones concerned condom-unprotected vaginal contacts (7:1), contacts in a state of alcohol intoxication of both partners (6:1) and contacts whenever only the student themselves was in a state of intoxication and when both partners were intoxicated with drugs (5:1).

### 3.2. Risky Sexual Experiences Clusters and Their Characteristics

**Figure 1:**
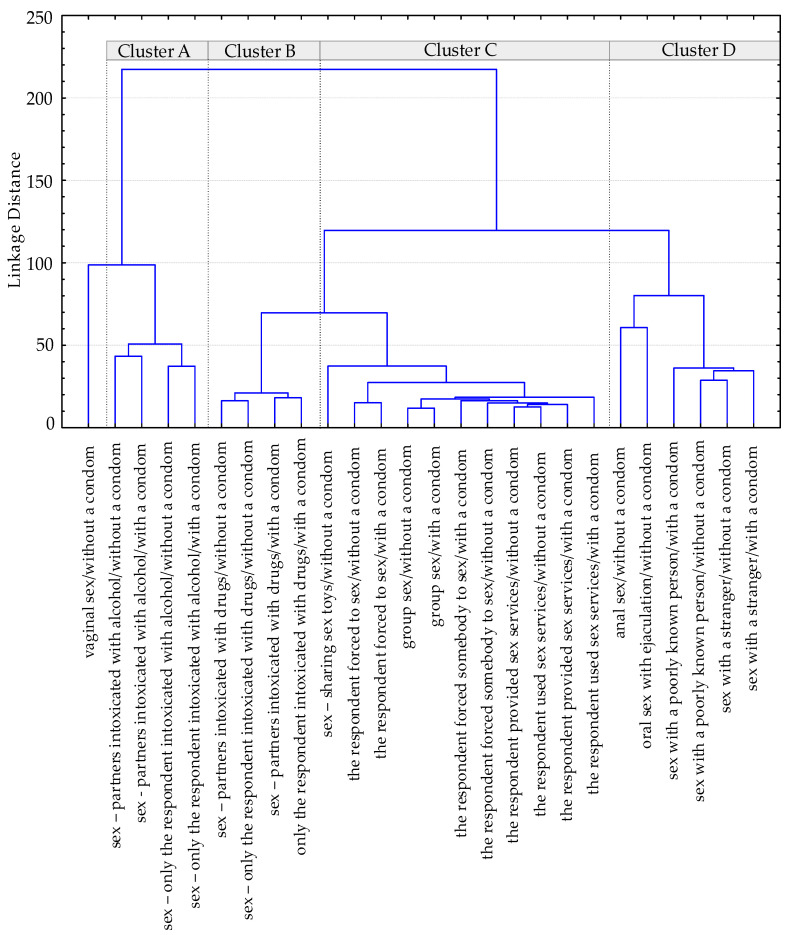
Risky sexual experiences clusters.

To date, detailed analysis has suggested that students form clusters of people who undertake similar risky behaviours. Therefore, in the second stage of the research, all the risky sexual experiences of students (divided into condom-protected and unprotected—26 behaviours—Figure 1) were analysed using the Ward’s method. It was stated that 60.3% of the respondents did not have risky experiences that would qualify them as belonging to the identified clusters, as opposed to the rest of the interviewees.

The resulting dendrogram (Figure 1) revealed the existence of four distinct clusters of students with similar risky experiences:

- A cluster—this encompasses persons undertaking unprotected or condom-protected sexual contacts, which took place while students themselves or students themselves and their partners were under the influence of alcohol (the dominant feature is alcohol intoxication of both partners); the behaviours of this group comprised 24% of the respondents;
- B cluster—this comprises persons undertaking unprotected or condom-protected sexual activities in a state of drug intoxication—of both students themselves and their partners (the dominant feature for this cluster is drug intoxication of both partners); the behaviours belonging to this cluster comprised 4.8% of the respondents;
- C cluster—this encompasses persons undertaking unprotected and condom-protected violent sexual contacts (where students play the role of both victims and perpetrators), providing and using paid sexual services, group sex, the use of shared, non-disinfected sex toys (the dominant feature for this cluster is the abuse of a partner and exceeding partner sexual norms); experiences gathered in this group comprised 3.1% of the respondents;
- D cluster—this concerns persons undertaking unprotected and condom-protected contacts with random, completely unknown or very little-known partners and condom-unprotected anal intercourse and oral contacts with ejaculation into the mouth (the dominant feature for this cluster is partners’ anonymity and going beyond the convention in sexual behaviours); the cluster comprised 17.8% of students.

The cluster analysis proved that the experience of unprotected vaginal contacts (undertaken by 39.7% of respondents) is an isolated behaviour and does not enter into any of the distinguished clusters, although it is risky.

### 3.3. Characteristics of Risky Sexual Experiences Clusters

In the third stage of the analysis, the author made an attempt to determine whether people who have similar risky sexual experiences distinguished within particular four clusters have similar features. For this purpose, the extent to which the 13 factors thatform the four groups (socio-demographic and self-independence ones, the way of fulfilling the role of a student, participating in social meetings/parties when intoxicated with psychoactive substances) mark out persons belonging to the isolated groups of risky behaviours.

A detailed analysis showed what percentage of students with specific features have risky sexual experiences distinguished within particular clusters (Table A2).

It was found that the risky sexual experiences isolated within the A cluster were declared in the highest percentage by the following persons:

- Heterosexuals with homosexual experiences (59.2%—out of the total number of heterosexual persons with homosexual experiences);
- People who declared having participated in numerous social meetings when intoxicated with drugs (55.5%);
- People who declared having participated in numerous social meetings when intoxicated with alcohol(44.5%);
- Non-believers and non-practitioners (36.7%);
- People whose parents were married but did not live together for months (34.4%) and whose parents were not married and never lived together (30.2%);
- People supported by family and persons from outside the family (33.3%);
- People who lived outside the family home for 5 years or more (30.9%);
- People who grew up in a big city with 100,000 residents or more (30.4%);
- People who are in a stable relationship or marriage (29.2%);
- Men (28.8%);
- Humanities students (27.8%);
- Students with the 3.5–3.9 grade averages in the previous term (27.7%);
- People in the age of 25 and older (27.5%).

Interestingly, the results show that homosexual people with heterosexual experiences and students repeatedly participating in social meetings when intoxicated with drugs exhibited these behaviours twice as often as the average for all the respondents (24%).

It was stated that risky sexual experiences isolated within the B cluster were declared in the highest percentage by the following persons:

- Homosexuals with heterosexual experiences (30.8% out of the group);
- Persons who repeatedly participated in social meetings when intoxicated with drugs (26.6%);
- Persons whose parents were not married but lived together all the time (17.1%) or were never married and never lived together (15.1%), non-believers who do not practice any religious rites at all (11.3%);
- Persons who grew up in a large city with over 100,000 inhabitants (10.1%);
- Persons supported by their family and other people from outside the family (10%);
- Persons who repeatedly participated in social meetings when intoxicated with alcohol (8.8%);
- Men (8%);
- Persons who have lived outside their family home for 5 years or more (7%);
- Students with the 3.5–3.9 grade averages in the previous term (6.7%);
- Humanities (6.4%) and medical and health sciences (5.7%) students;
- Persons aged 21–25 (5.5%);
- Singles (5.3%).

The research also showed that, out of all the respondents, the first six groups listed above engaged in behaviours identified within the present cluster in a percentage that ranged from two to six times higher than the average (4.8%).

It was revealed that the risky sexual experiences isolated within the C cluster were reported in the highest percentage by the following persons:

- Homosexuals with heterosexual experiences (23.1%), homosexuals (20%) and bisexuals (13.4%);
- Persons whose parents were never married and never lived together (11.3%);
- Persons who repeatedly participated in social meetings while intoxicated with drugs (9.5%);
- Non-believers, who participate in rituals for non-religious reasons (8.3%) and non-believers who do not practice at all (6.8%);
- Persons supported by the family and other people from outside the family (7.8%);
- Persons who have lived outside their family home for 5 years or more (6.3%);
- Persons who are over 25 years of age (5.8%);
- Men (5.8%);
- Students of arts (5.3%) and humanities (4.8%);
- Persons who grew up in a large city with over 100,000 inhabitants (5.1%);
- Students with the lowest academic performance (5%);
- Persons who repeatedly participated in social meetings while intoxicated with alcohol (4.9%);
- Singles (3.2%).

It should be emphasized that the first six groups of students listed above, characterised by specific features, undertake the behaviours identified within the present cluster in a percentage that ranges from two to seven times higher than the average for the research sample (3.1%).

It was stated that risky sexual experiences isolated within the D cluster were reported in the highest percentage by the following persons:

- Homosexuals with heterosexual experiences (46.2%) and homosexuals (42.9%);
- Persons who repeatedly participated in social meetings while being intoxicated with drugs (42.3%);
- Persons whose parents were never married and never lived together (37.7%);
- Students with the lowest results in the previous term (36.3%);
- Non-believers who do not practice at all (33.3%) and non-believers who participate in rites for non-religious reasons (28.3%);
- Men (27.2%);
- Students of agricultural sciences (26.6%);
- Persons who repeatedly participated in social meetings while being drunk (26.1%);
- Persons supported by the family and other people outside the family (25.6%) and who support themselves on their own (21.9%);
- Persons who have lived outside their family home for 5 years or more (24.3%);
- Persons brought up in a small city up to 20,000 residents (23.3%) or in a very large city with over 100,000 inhabitants (23%);
- Persons who are over 25 years of age (22.9%);
- Persons in a stable relationship or marriage (19%).

It was stated that the risky sexual experiences identified within the present cluster, in percentages of at least twice as high as the average for the research sample (17.8%), were undertaken by students from the first three subgroups enumerated above.

The analysis of the structure of particular four clusters (Table A3), due to the characteristics of people who have risky sexual experiences within the clusters, allowed for us to draw numerous conclusions.

As a result of the analyses, it was stated that, in comparison to the other clusters, among students undertaking risky sexual behaviours belonging to the A cluster (alcohol intoxication of partners as the dominant feature), the largest percentage are:

- Women—51.7% (percentage of women in the total number of women and men in the cluster; average for the remaining clusters is 32.9%; each time the value given in parentheses means the average for the remaining 3 clusters—marked as vs.);
- Persons up to 20 years old—18.7% (vs. 14.5%);
- Countryside inhabitants—47.7% (vs. 40.4%);
- Persons whose parents were married and lived together permanently—75.0% (vs. 64.5%);
- Heterosexuals—84.3% (vs. 75.5%) and asexuals—0.9% (vs. 0.3%);
- Believers who practice regularly—24.7% (vs. 19.2%);
- Persons living outside the family home for 3–4 years—20.2% (vs.14.4%);
- Persons supported only by their family—48.3% (vs.46.3%) or by their family and on their own—15.4% (vs. 11.6%);
- Persons in stable marriages or partnerships—64.3% (vs. 52.0%);
- Students of social sciences—42.7% (vs. 37.4%);
- Students with high grade averages: 4.5 and more—19.6% (vs. 16.2%);
- People who repeatedly participated in meetings under the influence of alcohol—49.9% (vs. 43.1%);
- People who never attended social meetings when intoxicated with drugs—60.8% (vs. 41.2%).

In comparison to the remaining clusters, among students having risky sexual experiences belonging to the B cluster (the predominant feature is drug intoxication of partners), the largest numbers are:

- Persons in the age of 21–26—64.2% (as opposed to the remaining clusters with the average 57.2%);
- Residents of large cities with over 100,000 inhabitants—19.4% (vs. 12.9%);
- Persons whose parents were married but did not live together—15.7% (vs. 12.7%) or whose parents were not married but lived together—5.2% (vs. 2.4%) or were divorced—11.2% (vs. 7.7%);
- Heterosexuals with homosexual experiences—10.4% (vs. 5.3%);
- Believers who do not practice any religious rites—18.7% (vs. 15.0%);
- Non-believers who do not practice at all—14.9% (vs. 11.9%) and the ones who find it difficult to declare their religious commitment—9.0% (vs. 5.8%);
- People who still live with their parents—40.3% (vs. 35.2%) or have lived alone for less than a year—17.2% (vs. 13.6%);
- People who do not live in stable intimate relationships—49.3% (vs. 39.7%);
- Students of medical sciences and health sciences—21.6% (vs. 18.5%), and exact and natural sciences students—5.2% (vs. 3.7%);
- Students with low academic performance: grade averages below 3.5—6.7% (vs. 4.3%) or from 3.5 to 3.9—32.8% (vs. 29.5%);
- Persons who participated in social meetings under the influence of alcohol no more than twice—28.4% (vs. 27.3%);
- People who participated once or twice in social meetings while intoxicated with drugs—27.6% (vs. 18.9%) or had multiple experiences of this type—54.5% (vs. 25.4%).

In comparison to the remaining clusters, among students with risky sexual experiences belonging to the C cluster (whose dominant feature is the abuse of one of the partners and exceeding partner sexual norms), the highest percentages are:

- Men—73.6% (vs. 58.7%);
- People aged 26 and more—17.2% (vs. 10.8%);
- Residents of cities with 20,000–100,000 inhabitants—21.8% (vs. 20.2%);
- Bisexual people—10.3% (vs. 6.6%);
- People whose parents have never been married and have never lived together—6.9% (vs. 4.1%);
- Homosexual persons with heterosexual experiences—3.4% (vs. 1.7%) and homosexual persons—8.0% (vs. 3.2%);
- People who are deeply religious and participate in rituals regularly—11.5% (vs. 5.7%);
- Persons who do not believe but practice for non-religious reasons—5.7% (vs. 3.1%);
- Persons who have lived outside their family home for 1–2 years—20.7% (vs. 16.6%) or longer than 5 years—19.5% (vs. 13.4%);
- People supported by both family and unknown persons—8.0% (vs. 5.3%);
- Students of humanities—10.3% (vs. 8.0%) and arts—3.4% (vs. 1.9%).

In comparison to the remaining clusters, among students with risky sexual experiences belonging to the D cluster (the dominant feature of this cluster is partners’ anonymity and going beyond convention in sexual behaviour), the largest percentages are:

- Residents of small cities up to 20,000 inhabitants—24.9% (vs. 20.8%);
- People whose one or both parents were dead—3.0% (vs. 1.7%);
- Persons who believe but practice irregularly—35.1% (vs. 31.4%);
- Self-independent persons who support themselves on their own—36.7% (vs. 33.3%);
- Students of engineering and technical sciences—26.0% (vs. 22.9%) and agricultural sciences—6.9% (vs. 2.3%);
- People who have never participated in social meetings under the influence of alcohol—32.9% (vs. 25.0%).

## 4. Discussion

When analysing risky sexual behaviours, it is worth noting that the generation of young Poles, including students, has significant deficits in terms of sex education. Population studies show that 31% of the respondents (at the age of 18–26 years old) did not have sex education at school or found it useless, and 42% never spoke to their parents about sexuality [60]. The basic source of knowledge of the sexuality and sexual behaviour patterns of modern young adults are mainly peers, the Internet, especially Internet forums, portals, blogs, films and magazines, including erotic and pornographic ones [40,61] and series (e.g., the Netflix series ‘Sex Education’ [62]). The consequences of these phenomena are observed in the intensive changes in the young generation’s sexual morality [63]. The educational deficits result, inter alia, in negative attitudes towards the use of condoms [64] and resignation from their use [52].

Speaking of university students’ risky sexual behaviours, attention should be paid to the great technological and communication changes that have taken place in Poland. The popularization of mobile telephony, the Internet [65] and the increased possibilities of moving around the country and the world, combined with students’ social competences, foster the establishing of numerous social and intimate relationships (e.g., via dating applications [66]). Moreover, it should not be forgotten that being in the university environment itself also gives many opportunities to engage in various risky behaviours, including sexual ones [29,67].

Three stages of the analysis were carried out in the presented material, and they allowed us to solve the following research problems:

- What is the prevalence and forms of students’ risky sexual experiences?
- Do students form clusters similar to each other in terms of risky sexual experiences, and if so, what are they like?
- Which of the studied factors characterise and mark out the respondents with particular risky sexual experiences in terms of the defined clusters of risky sexual behaviours?

### 4.1. The Prevalence and Forms of Students’ Risky Sexual Experiences

The collected results prove that a high percentage of students from the Podkarpacie region have experienced risky sexual behaviours, especially condom-unprotected vaginal, oral, anal, contacts, and both condom-protected and unprotected contacts in a state of alcohol intoxication of partners and intercourse with strangers or poorly known persons.

The comparison of the prevalence of risky experiences of students of the Podkarpacie region (details below), along with the results of other Polish and global research carried out in academic circles, suggests (limited conclusions due to methodological differences) that they undertake unprotected vaginal contacts and have intercourse in a state of intoxication with alcohol more often than other students. However, the prevalence of declared activity with strangers, as well as providing and using paid sexual services, is similar. When it comes to engaging in sexual activity while intoxicated with drugs, violent contacts and group contacts, the authors’ research revealed lower rates than in other studies from Poland and worldwide.

It was stated that a high percentage of students from Podkarpacie universities have numerous risky sexual experiences. These are mainly: vaginal contacts not protected with a condom (almost 40% in total), oral contacts with ejaculation into the mouth (almost 22%) and anal ones (almost 14%). Consistent use of condoms during vaginal contacts was declared by 23%, during anal contacts—by 24%, and oral contacts—by only 11% of the respondents who ever undertook this type of activity. A comparison with the results of other Polish and worldwide studies shows that students of the Podkarpacie region are much less consistent in the use of condoms when undertaking vaginal contacts, and more consistent when it comes to anal and oral contacts. At the same time, similarly to participants in other studies, they use condoms more often during vaginal contacts than during oral contacts with ejaculation, which they probably consider safe.The aversion to condoms and, in particular, the extreme inconsistency in their use presented by the respondents, is in line with the attitudes of Poles in general. The population research performed by Izdebski shows that, in the year preceding the survey, the following percentages of sexually active Poles never used a condom: 39.9% during vaginal intercourse, 62.5% during anal intercourse, and 77.1% during oral intercourse [40]. The trend of intercourse without condom protection recorded in the author’s research confirms previous studies carried out in groups of students, both in Poland [19,22,68] and other countries [23,24,25,26,28]. They show that consistent use of condoms during vaginal intercourse was declared by no more than 60% of students, during anal intercourse by 5–30%, and oral contacts by no more than 6%.

The analyses of the author’s research have shown that a high percentage of students from Podkarpacie universities have experiences of sexual intercourse when they were under the influence of alcohol (almost 21%) or when they were both intoxicated with their partners (28%). In addition, nearly 80% of them undertook the activities without condom protection. For comparison, nationwide studies of students show that 27% of them had ever had sex under the influence of alcohol [22]. Interestingly, a meta-analysis by Hingson showed that the rate of American students who had unprotected intercourse as a result of being intoxicated with alcohol was much lower—around 8% [69].

Research on Podkarpacie students has revealed that over 5% of them had experiences of sexual contacts while intoxicated with drugs. It should be emphasised that in some groups of respondents, e.g., homosexuals with heterosexual experiences, experiences of sexual intercourse in a state of intoxication with psychoactive drugs concerned half of the respondents. This result corresponds with the findings from the EMIS 2017 research carried out in the group of MSM Poles, which shows that 12% had experiences of sexual intercourse during which they were sexually stimulated with drugs or alcohol [9].

On the basis of the author’s analyses, it can be stated that a high percentage of respondents have experience of contacts with complete strangers (almost 14%) or very little-known persons (almost 17%). Additionally, about 60% of them said they also had unprotected sex.

During the implementation of the research, a group of students from the University of Pristina, Kosovo, also revealed a high percentage of people who did not use a condom during intercourse with an unknown partner—only 56.8% reported using a condom consistently [25]. Population studies of students in Poland show that 19% of them have experience of ‘casual sex’ [22], so the ratio relating to students of the Podkarpacie region is slightly lower.

During the present research, it was found that several percent of students from Podkarpacie universities are involved in commercial sexual activity. The percentage is similar to the results obtained in studies performed on students in other countries.

A total of 2.1% of the respondents admitted to having provided sexual services. Nevertheless, the ratio is lower than the one obtained in another Polish study of students (conducted online), in which 17.2% of respondents declared having provided paid sexual services, and 10.4% other sponsorship services [32]. The results of the author’s research are similar to those obtained in other European countries, e.g., in Great Britain [33] or Germany [34] cf. [35], because, although the percentage of people declaring the provision of various sexual services was 6–7%, sexual intercourse accounted for about 0.2% of the behaviours; thus, it concerned 1–2% of the respondents. The Student Sex Work Project carried out in 2015 at the University of Swansea (2015) shows that 5% of the respondents were active in the sex industry, while only some of them had sexual contacts with clients [36].

The research on the group of Podkarpacie students found that they both provide and use sexual services. The use of paid sexual services was confirmed by 3.2% of the respondents. On the basis of the available reports, it is estimated [70] that, worldwide, such services are used by 2% of young women and 6% of young men; however, the figures differ slightly. For example, Canadian research shows that about 2% of young women and about 5% of young men use sexual services [71], and according to a Swiss study, 0% of women and 5.4% of young men, respectively [70].

The results of the author’s study showed that almost 3% of the respondents experienced group sex; however, this is a much lower indicator than in other available studies. For comparison, during previous research on Polish students, it was found that group sex concerned 10.1% of the total [72].

According to American reports, 13% of young adults had sexual experiences in a ‘three-way’ [73].

The author’s analyses showed that 3.7% of the respondents were forced into unwanted relationships. A study carried out on American students shows that the percentage of students who have experienced sexual assault is much higher and amounts to 13% [37]. The fact that 2.6% of Podkarpacie students declared that they had used sexual violence is particularly problematic. However, this is a relatively low result in the context of previous research performed by Waszyńska, which revealed that 5.4% of students initiated sexual behaviour towards people under the age of 15 [38].

The research revealed that among people who engage in various risky sexual behaviours, the majority (60–87%) do not consistently use condoms. The obtained results accurately justify the epidemiological situation in Poland, where, in the past decade, people aged 20–29 accounted for one third of newly detected HIV infections [8,15]. Additionally, in 2020, Poles (both women and men) aged 20–29 developed syphilis, gonorrhoea and Chlamydia [74] more than three times more often than the population average. Previous nationwide studies among students show that 5% of them suffered from a sexually transmitted disease [22]. It should be emphasized that the risk of contracting sexually transmitted infections to the health of the young generation is exacerbated by Poles’ strong resistance to making diagnoses. The percentage of people testing themselves ranges from 4% in the 18–19 age group to 16% in the 40–49 age group [40].

When it comes to the consequences for sexual, reproductive and mental health, the majority of students from the Podkarpacie region much more often declared repeated risky sexual behaviours than one-off experiences. The phenomenon suggests that the disordered sex script has been consolidated (cf. [57,58]) and the respondents will continue to follow the learnt behaviour model in the future. According to the concept of sexual scripting, behaviour models that were once learnt at the individual level tend to persist and pararitualize. The script is consolidated, especially when a found behaviour formula makes it possible to achieve sexual satisfaction and acquire socio-sexual competences [75].

### 4.2. Clusters of Risky Sexual Experiences and Their Characteristics

The analysis carried out using Ward’s method enabled us to distinguish four clusters of students who have similar risky sexual experiences. It was also found that 60.3% of the respondents do not form isolated clusters. The highest percentage of students (24%) undertook behaviours, which, together, are marked as the A cluster. Its distinguishing feature was establishing sexual relations under the influence of alcohol. A large cluster (including 17.8% of students) marked as the D cluster encompassed sexual behaviours characterised by the anonymity of sexual partners and transgressing the convention. There were also much smaller clusters such as the B cluster (4.8% of respondents; the distinguishing feature was the drug intoxication of sexual partners) and the C cluster (3.1% of students; the dominant feature was partner abuse and exceeding partner’s sexual norms).

It should be noted that each of the above-mentioned groups of behaviours is associated with slightly different risks for the respondents. The A and B clusters should primarily be considered through the prism of the mechanisms resulting from the influence of alcohol and drugs. In this case, the risk determinants are mainly disorder of cognitive processes resulting in the focus on the advantages of sexual activity and neglecting threats (cf. ‘alcohol myopia’) [76,77,78], lower cognitive reserve, which means problems with making decisions that require a complex analysis of the potential gains and losses resulting from sexual activity [79], problems with an adequate assessment of the risk resulting from a sexual contact, increased readiness to enter into a risky sexual relationship and engaging in behaviours with the highest risk level, willingness to overcome one’s own sexual barriers [80,81,82], changes in experiencing excitement, desire and orgasm under the influence of psychoactive substances [83], changes in sexual reactivity, especially arousal and inhibition [76,84], changes in the assessment of potential sexual partners’ attractiveness, which contributes to greater openness to intercourse, even with random persons [85], tendency to select partners on impulse [86,87], unreasonable resistance to using condoms [19,26,38] or problems with their proper use [87,88,89], increase in the tendency to resort to sexual aggression [90,91,92].

As far as sexually transmitted infections (STI) are concerned, the tendency of intoxicated people to give up using condoms (even during sexual contact with a newly met person) is particularly important. It has been proved many times by various studies that alcohol consumption increases the probability that a person will not use a condom during sexual activity [93]. An unjustified resistance to its use was observed, especially in men. This objection is expressed both in not using condoms [94], and greater willingness to have unprotected sex [77,95,96,97]. Condom-use resistance (CUR) can be defined as behaviour that strategically aims at having sexual intercourse without a condom in the situation when the intimate partner wants to use it and it is possible to do so, e.g., there is a condom available [98]. Such behaviour should be, therefore, treated as a specific form of sexual aggression that occurs after the use of alcohol [92].

It is particularly important to underline that intoxication with psychoactive substances coexists with sexual aggression [99,100,101,102,103]. The thesis has been confirmed many times in world studies, especially those concerning alcohol abuse [90,91,92].

As a result, the above-mentioned effects of intoxication with alcohol or other psychoactive substances may result in a random (even unacceptable in a state of sobriety) choice of an intimate partner, accidental (even unacceptable) circumstances of intercourse, previously unacceptable forms of intercourse, the unpredictability of one’s own or partner’s/partners’ reactions, neglecting one’s own and partners’ safety when it comes to undertaking dangerous forms of intercourse, or not using protection against unwanted pregnancy or sexually transmitted infections, mechanical damage to the body resulting from violent reactions stimulated by psychoactive substances, undertaking forms of intercourse which violate legal norms (e.g., rape, intercourse with minors, with animals).

Threats related to having sexual experiences from the C cluster can be treated as cumulative. They are potentially associated with sexual contacts with many people of unknown serological status, sexual preferences and personality features (including personality disorders). Therefore, they entail an increased risk of sexually transmitted diseases, experiences of various forms of violence, criminal behaviour, substance abuse, and mental and physical health problems [104,105,106]. According to the literature analysis, they often coexist with the use of alcohol or other psychoactive substances. For example, it was found that 40–90% of rape perpetrators committed the crime while intoxicated with alcohol [107]. As Abbey states, half of college students experienced a sexual assault in a situation when they were under the influence of alcohol, when the perpetrator was so or when both the respondent and the perpetrator were intoxicated with alcohol [108]. The previously cited meta-analysis by Hingson shows that as a result of alcohol intoxication, 2% of the interviewees were rape victims, including rape on a date [69]. The research conducted among college female students shows that, of all of them who have experienced rape, 72% occurred when the victim was too intoxicated to express conscious consent or objection [109].

The threats associated with the experiences assigned to the D cluster are primarily related to the risk of contracting a sexually transmitted infection, unplanned pregnancy, the problem of finding a partner in case of the resulting consequences, and especially the feeling of regret and disappointment because of accidental intercourse with an unknown person [53], establishing an intimate relationship with a partner whom a person normally does not desire [54], not using a condom and anticipated damages resulting from this neglect [56].

The analysis proved that the features that distinguish persons belonging to the four defined clusters of risky sexual experiences are: sexual orientation, participation in social meetings/parties under the influence of drugs or alcohol, religious commitment, respondents’ parents’ relationship model and gender.

The presented material relates, to a large extent, to public health problems, primarily to students’ sexual and reproductive health. The IPPF Charter on Sexual and Reproductive Rights states that, in relation to persons under 25 years of age, sexual and reproductive health means physical and emotional well-being related to being healthy and free from premature and unwanted pregnancy, unsafe abortion, and sexually transmitted infections including HIV/AIDS, as well as sexual violence and coercion [110]. The obtained results prove that, due to the prevalence, type, and repeatability of risky sexual experiences and their clusters, students from the Podkarpacie region are a group who is exposed to the negative consequences of the behaviours—primarily to sexual and reproductive health, but also somatic and mental consequences.

The discussed experiences of the studied population suggest, above all, a high risk of infection with sexually transmitted diseases, including HIV infections, and unwanted accidental pregnancies (as a result of the lack of condoms and random sexual partners), bodily injuries, including those resulting from sexual violence (as a result of intoxication with psychoactive substances of both partners and contacts with unknown partners), lowered mood, self-esteem, sense of regret (due to undertaking risky sexual activity, choice of a random partner and anticipation of future consequences) and the possibility of becoming a crime victim, including crimes other than sexual ones. It should be also noted that the repeatability of risky sexual behaviours, which prove that they constitute a fixed sexual script among the respondents, significantly increases the probability of any negative consequences.

In the interest of public health, it is, therefore, indispensable to intensify efforts in the field of sex education, which, as has been repeatedly proven (e.g., in studies carried out among British students [111]), significantly reduces the risk of engaging in unprotected sexual intercourse and being contracted with sexually transmitted diseases. However, not only do the efforts have to focus on the transfer of knowledge (related to psychosexual development, sexually transmitted diseases—especially in terms of the possibilities and procedures of diagnosis and treatment, contraception, including post-coital contraception, risky sexual behaviours—their characteristics and consequences), but, above all, on shaping the desired attitudes of students and social skills [112]. The key issue (which is generally overlooked in Polish sex education [113]) is the fact that all interactions should consider the actual experiences and needs of students, and not social stereotypical perceptions on the matter.

From the perspective of the diagnosed prevalence of students’ risky sexual behaviours, and especially the knowledge of their accumulation in clusters of risky experiences, it is necessary to direct the impacts to the entire community of students, and not to a selected group of activists. The optimal goal of student sex education would be a complete elimination of all risky behaviours. However, an adequate and realistic goal seems to be focus on developing competences related to risk assessment and management in sexual situations [114], which would enable the effective avoidance or minimization of sexual risk. In addition, students should also be equipped with knowledge about the possibilities and ways of coping with a situation when a risky sexual activity has already occurred.

According to the analyses, the majority of students who have risky sexual experiences belong to the A cluster, with alcohol intoxication of partners as a dominant feature, and the D cluster (17.8%), whose distinguishing feature is partners’ anonymity and going beyond conventional behaviours. In this context, one of the most important postulates relating to students’ sex education is the integration of risky sexual behaviour problems with the abuse or addiction to psychoactive substances and the tendency to seek sensations, cf. [115].

### 4.3. Research Weaknesses and Strengths, Future Directions

Research weaknesses:

- In the current research, 13 determinants of risky sexual behaviours were taken into account. In the next round of research it would be necessary to consider more determinants, especially experiences of child abuse and neglect, attachment style, tendency to seek sensations;
- In the research, students were asked about any risky sexual experiences they ever had. In the future, it would be worth checking experiences that took place only during the studies;
- The study was carried out in a very traditional region of the country, so it would be important to repeat the research on the nationwide student population;
- Since the study in 2019, major changes have taken place in the Podkarpacie region resulting from the COVID-19 pandemic and the war in Ukraine, so it seems important to repeat the research in a completely new social situation.

Research strengths:

- An innovative grouping of questions concerning sexual experiences was applied according to two situations: with and without the use of a condom;
- The cluster analysis allowed for us to define four different styles of students’ risky functioning in the sexual sphere;
- The research covered students from all universities in the Podkarpacie region;
- It was conducted in direct contact with the respondents, which made it possible to collect answers from the real class of students and excluded the possibility of filling in the questionnaires by random people (as happens in online research);
- Thanks to the face-to-face research, it was possible to provide the respondents with information support in the form of educational materials on research issues, contact details of aid institutions and specialist’s advice (a sex educator) after the survey.

## 5. Conclusions

It has been proved that a high percentage of students from universities in the Podkarpacie region have various and multiple risky sexual experiences, most often, they are the following: unprotected vaginal, oral, anal intercourse, sex under the influence of alcohol as well as intercourse with strangers or with very little known people. A characteristic feature is a fact that, even in relation to the most risky sexual contacts, e.g., with strangers or when providing or using sexual services, the respondents use condoms inconsistently. It was found that, in the group of interviewees undertaking risky behaviours, up to 87% had experiences of contacts not protected with a condom.

The respondents form four clusters of people having similar risky experiences: most often the A cluster (24%), with alcohol intoxication of partners as a dominant feature, and the D cluster (17.8%), whose distinguishing feature is partners’ anonymity and going beyond conventional behaviour. It was found that belonging to particular clusters was mainly related to sexual orientation, social experiences under the influence of drugs or alcohol, religious commitment, or gender.

As the obtained results show, it is necessary to implement sex-education programs focused on developing competences related to avoiding or minimizing risks in sexual situations and equipping students with knowledge that can reduce the negative consequences of risky behaviours. It is also essential to integrate the subject of risky sexual behaviours with the abuse of psychoactive substances and the tendency to seek sensations.

## Figures and Tables

**Table 1:** Characteristics of the research sample, *n* = 2764.

Main Category of Independent Variables	Subcategory of Independent Variables	Response Category	Percentage in the Research Sample *
Socio-demographic factors	Gender	Woman	59.7
		Man	40.3
	Age	Up to 20 years old	22.3
		21—25 years old	56.7
		26 years old and more	9.3
	Place of growing up	Countryside	54.3
		A town with 20,000 inhabitants or less	19.1
		A town with 20,000–100,000 inhabitants	16.6
		A town with more than 100,000 inhabitants	9.3
	Parents’ relationship model	Married parents who permanently lived together	78.7
		Married parents who did not live together for months	7.9
		Unmarried parents who permanently lived together	1.5
		Unmarried parents who never lived together	1.9
		Divorced or separated parents	6.1
		One or both parents dead	2.4
		Another situation	0.4
	Sexual orientation	Only heterosexuals	89.3
		Heterosexuals with homosexual experiences	2.6
		Bisexuals	2.4
		Homosexuals with heterosexual experiences	0.5
		Only homosexuals	1.3
		Asexuals	1.6
	Religious commitment	Deeply religious persons who practice regularly	11.3
		Religious persons who practice regularly	33.3
		Religious persons who practice irregularly	28.6
		Religious persons who do not practice at all	11.7
		Non-believers who practice	2.2
		Non-believers who do not practice	6.4
		Hard to say	5.4
Self-independence determinants	Period of living outside the family home	Still living with parents or legal guardians	37.4
		Less than a year	17.9
		1–2 years	15.9
		3–4 years	17.9
		5 years or more	9.8
	Student’s support sources while studying	Only family	51.8
		Family and persons outside the family	3.3
		Student: themselves and family and (or) persons outside the family	13.9
		Only student themselves	29.8
	Living in a stable partner relationship or marriage	Yes	52.7
		No	44.7
Ways of fulfilling the role of a student	Studied field of science	Humanities	6.8
		Engineering and technical sciences	24.0
		Medical and health sciences	18.5
		Agricultural science	4.6
		Social science	39.3
		Exact and natural sciences	4.8
		Arts	2.1
	Average of the grades received in the previous term	Less than 3.5 **	2.9
		3.5–3.9	23.8
		4.0–4.4	47.0
		4.5 and more	24.1
Attending social meetings/parties while intoxicated with psychoactive drugs	Attending social meetings/parties under the influence of alcohol	No	46.6
		Yes, once or twice	25.3
		Yes, 3 times or more	26.8
	Attending social meetings/parties while intoxicated with drugs	No	78.6
		Yes, once or twice	10.0
		Yes, 3 times or more	9.9

* The results were calculated with reference to the entire sample, differences up to 100% indicate gaps in the answers provided. ** In the Polish higher education system, the grading scale is 2.0–5.0.

**Table 2:** Students’ risky sexual experiences, *n* = 2764 (in %).

	Frequency				Experiences in Total
Risky Sexual Experiences	Never	Once	More Than Once	No Answer	
1. Sexual contact with a stranger—in total	80.0	4.5	9.1	6.4	13.6
1.1. with a condom	81.9	6.0	6.0	6.0	12.1
1.2. without a condom	84.7	4.8	3.5	7.0	8.3
2. Sexual contact with a poorly known person—in total	76.9	6.2	10.3	6.5	16.5
2.1. with a condom	79.2	8.2	6.2	6.4	14.4
2.2. without a condom	83.3	5.4	4.2	7.2	9.6
3. Oral contact with ejaculation into the mouth—in total	68.5	4.1	20.4	7.1	24.5
3.1. with a condom	80.6	3.7	8.3	7.4	12.0
3.2. without a condom	71.0	4.8	16.9	7.3	21.7
4. Vaginal sex—in total	41.9	3.2	48.5	6.4	51.7
4.1. with a condom	45.1	5.5	42.6	6.7	48.1
4.2. without a condom	52.9	5.1	34.6	7.4	39.7
5. Anal sex—in total	74.9	4.0	13.9	7.1	17.9
5.1. with a condom	79.1	4.6	9.1	7.2	13.7
5.2. without a condom	79.2	4.3	9.2	7.3	13.6
6. Sharing erotic toys—in total	86.6	1.7	5.0	6.7	6.7
6.1. with a condom	88.7	2.1	2.9	6.4	4.9
6.2. without a condom	88.1	2.2	2.9	6.8	5.1
7. Using sexual services (oral, vaginal or anal contacts)—in total	90.1	0.9	2.3	6.6	3.2
7.1. with a condom	91.0	1.4	1.4	6.2	2.8
7.2. without a condom	91.2	1.1	1.1	6.6	2.2
8. Providing sexual services (oral, vaginal or anal contacts)—in total	91.2	0.5	1.6	6.6	2.1
8.1. with a condom	92.2	0.6	0.9	6.3	1.5
8.2. without a condom	91.6	0.8	0.9	6.7	1.7
9. Group sex—in total	90.6	1.1	1.7	6.5	2.8
9.1. with a condom	91.6	1.3	1.1	6.1	2.3
9.2. without a condom	91.5	0.8	1.1	6.7	1.9
10. Coercing somebody to sexual contact—in total	90.7	0.5	2.1	6.6	2.6
10.1. with a condom	91.6	0.9	1.3	6.2	2.1
10.2. without a condom	91.5	0.8	1.0	6.7	1.8
11. Being coerced to sexual contact—in total	89.6	1.1	2.6	6.7	3.7
11.1. with a condom	90.9	1.4	1.5	6.3	2.9
11.2. without a condom	90.4	1.2	1.7	6.7	2.9
12. Sexual contact when only the student was under the influence of alcohol	72.6	3.6	17.3	6.6	20.9
12.1. with a condom	75.2	5.1	13.3	6.3	18.5
12.2. without a condom	78.4	3.8	11.0	6.9	14.8
13. Sexual contact when both partners were under the influence of alcohol	65.3	4.2	24.1	6.4	28.3
13.1. with a condom	68.1	7.1	18.4	6.4	25.5
13.2. without a condom	72.7	4.8	15.5	7.0	20.3
14. Sexual contact when only the student was intoxicated with drugs	88.1	1.2	4.2	6.5	5.4
14.1. with a condom	89.0	2.0	2.8	6.2	4.8
14.2. without a condom	89.5	1.3	2.7	6.5	4.0
15. Sexual contact when both partners were intoxicated with drugs	88.2	0.9	4.4	6.5	5.3
15.1. with a condom	89.2	1.5	3.2	6.2	4.7
15.2. without a condom	89.2	1.3	2.9	6.6	4.2

## Data Availability

The data are owned by University of Rzeszów and are not to be made freely publicly available.

## Appendix A

**Table A1:** Examined sexual behaviours.

Sexual Experiences	Variable Category
Sexual contact with a complete stranger:	Never
With a condom	Once
Without a condom	More than once
Sexual contact with a poorly known person:	Never
With a condom	Once
Without a condom	More than once
Oral contact with ejaculation into the mouth:	Never
With a condom	Once
Without a condom	More than once
Vaginal sex:	Never
With a condom	Once
Without a condom	More than once
Anal sex:	Never
With a condom	Once
Without a condom	More than once
Sharing erotic toys:	Never
With a condom	Once
Without a condom	More than once
Using sexual services (oral, vaginal or anal contacts):	Never
With a condom	Once
Without a condom	More than once
Providing sexual services (oral, vaginal or anal contacts):	Never
With a condom	Once
Without a condom	More than once
Group sex:	Never
With a condom	Once
Without a condom	More than once
Coercing somebody to sexual contact:	Never
With a condom	Once
Without a condom	More than once
Being coerced to sexual contact:	Never
With a condom	Once
Without a condom	More than once
Sexual contact when only the student was under the influence of alcohol:	Never
With a condom	Once
Without a condom	More than once
Sexual contact when both partners were under the influence of alcohol:	Never
With a condom	Once
Without a condom	More than once
Sexual contact when only the student was intoxicated with drugs:	Never
With a condom	Once
Without a condom	More than once
Sexual contact when both partners were intoxicated with drugs:	Never
With a condom	Once
Without a condom	More than once

**Table A2:** Characteristics of the identified clusters (% out of particular response category) (*n* = 2764).

Main Category of Independent Variables	Detailed Category	Response Category	Clusters of Risky Sexual Experiences *				
			A *n* = 663	B *n* = 134	C *n* = 87	D *n* = 493	Not Belonging to Any Cluster *n* = 1667
Socio-demographic factors	Gender	Woman (*n* = 1651)	20.8	2.7	1.4	11.5	65.5
		Man (*n* = 1113)	28.8	8.0	5.8	27.2	52.6
	Age	Up to 20 years old (*n* = 616)	20.1	2.8	2.1	12.7	64.0
		21–25 years old (*n* = 1566)	26.5	5.5	2.9	18.0	58.3
		26 years old and more (*n* = 258)	27.5	5.0	5.8	22.9	56.6
		No answer (*n* = 324)	16.4	5.6	4.3	22.8	66.0
	Place of growing up	Countryside (*n* = 1502)	21.0	3.3	2.5	13.8	65.1
		A town with 20,000 inhabitants or less (*n* = 528)	26.5	5.5	3.2	23.3	55.9
		A town with 20,000–100,000 inhabitants (*n* = 459)	27.5	6.3	4.1	21.6	53.4
		A town with more than 100,000 inhabitants (*n* = 257)	30.4	10.1	5.1	23.0	53.7
		No answer (*n* = 18)	16.7	5.6	5.6	22.2	61.1
	Parents’ relationship model	Married parents who permanently lived together (*n* = 2178)	22.8	3.7	2.6	15.7	62.1
		Married parents who did not live together for months (*n* = 218)	34.4	9.6	5.5	29.4	50.0
		Unmarried parents who permanently lived together (*n* = 41)	19.5	17.1	7.3	31.7	46.3
		Unmarried parents who never lived together (*n* = 53)	30.2	15.1	11.3	37.7	41.5
		Divorced or separated parents (*n* = 169)	26.0	8.9	5.3	17.8	59.8
		One or both parents dead (*n* = 66)	24.2	3.0	1.5	22.7	59.1
		Another situation (*n* = 11)	27.3	9.1	0.0	18.2	45.5
		No answer (*n* = 28)	14.3	0.0	0.0	21.4	67.9
	Sexual orientation	Only heterosexuals (*n* = 2468)	22.6	3.8	2.6	16.4	61.3
		Heterosexuals with homosexual experiences (*n* = 71)	59.2	19.7	4.2	42.3	26.8
		Bisexuals (*n* = 67)	44.8	19.4	13.4	40.3	40.3
		Homosexuals with heterosexual experiences (*n* = 13)	46.2	30.8	23.1	46.2	46.2
		Only homosexuals (*n* = 35)	37.1	17.1	20.0	42.9	34.3
		Asexuals (*n* = 44)	13.6	2.3	0.0	2.3	86.4
		No answer (*n* = 66)	10.6	1.5	1.5	15.2	77.3
	Religious commitment	Deeply religious persons who practice regularly (*n* = 313)	12.1	2.9	3.2	7.3	78.6
		Religious persons who practice regularly (*n* = 920)	17.8	1.8	2.3	11.2	69.5
		Religious persons who practice irregularly (*n* = 790)	28.6	5.7	2.9	21.9	53.2
		Religious persons who do not Practice at all (*n* = 324)	31.8	7.7	3.7	23.8	50.6
		Non-believers who practice (*n* = 60)	23.3	8.3	8.3	28.3	48.3
		Non-believers who do not practice (*n* = 177)	36.7	11.3	6.8	33.3	41.2
		Hard to say (*n* = 150)	32.7	8.0	2.0	22.0	50.7
		No answer (*n* = 30)	13.3	3.3	3.3	26.7	66.7
Self-independence factors	Period of living outside the family home	Still living with parents or legal guardians (*n* = 1035)	22.7	5.2	2.7	18.1	61.9
		Less than a year (*n* = 496)	21.0	4.6	1.6	15.9	62.3
		1–2 years (*n* = 440)	23.4	5.2	4.1	19.3	58.6
		3–4 years (*n* = 496)	27.0	3.0	3.0	14.7	60.3
		5 years or more (*n* = 272)	30.9	7.0	6.3	24.3	52.2
		No answer (*n* = 25)	12.0	0.0	4.0	12.0	72.0
	Student’s support sources while studying	Only family (*n* = 1431)	22.4	4.5	2.8	15.6	63.6
		Family and persons outside the family (*n* = 90)	33.3	10.0	7.8	25.6	43.3
		Student-themselves and family and (or) persons outside the family (*n* = 384)	26.6	3.9	2.6	15.4	57.3
		Only student-themselves (*n* = 825)	25.0	5.6	3.6	21.9	57.7
		No answer (*n* = 34)	14.7	0.0	0.0	20.6	64.7
	Living in a stable partner relationship or marriage	Yes (*n* = 1457)	29.2	4.5	3.0	19.0	51.3
		No (*n* = 1235)	18.1	5.3	3.2	16.2	70.6
		No answer (*n* = 72)	18.1	2.8	5.6	22.2	65.3
Ways of fulfilling the role of a student	Studied field of science	Humanities (*n* = 187)	27.8	6.4	4.8	18.7	56.1
		Engineering and technical sciences (*n* = 663)	23.2	4.1	3.3	19.3	61.1
		Medical and health sciences (*n* = 510)	24.5	5.7	2.9	18.8	59.2
		Agricultural science (*n* = 128)	15.6	1.6	1.6	26.6	59.4
		Social science (*n* = 1087)	26.0	5.0	3.0	15.4	60.8
		Exact and natural sciences (*n* = 132)	17.4	5.3	2.3	15.9	63.6
		Arts (*n* = 57)	10.5	5.3	5.3	21.1	59.6
	Average of the grades received in the previous term	Less than 3.5 (*n* = 80)	20.0	11.3	5.0	36.3	48.8
		3.5–3.9 (*n* = 657)	27.7	6.7	3.8	24.2	53.9
		4.0–4.4 (*n* = 1300)	25.1	4.8	3.3	16.2	60.7
		4.5 and more (*n* = 666)	19.5	2.7	2.3	13.4	66.1
		No answer (*n* = 61)	14.8	1.6	0.0	9.8	73.8
Attending social meetings/parties while intoxicated with psychoactive drugs	Attending social meetings/parties under the influence of alcohol	No (*n* = 1287)	11.7	2.3	2.0	12.6	73.0
		Yes, once or twice (*n* = 698)	25.8	5.4	3.4	19.2	56.9
		Yes, 3 times or more (*n* = 742)	44.6	8.8	4.9	26.1	39.9
		No answer (*n* = 37)	2.7	2.7	2.7	8.1	91.9
	Attending social meetings/parties while intoxicated with drugs	No (*n* = 2172)	18.6	1.1	1.9	13.3	66.5
		Yes, once or twice (*n* = 277)	38.6	13.4	7.2	31.0	40.1
		Yes, 3 times or more (*n* = 274)	55.5	26.6	9.5	42.3	27.4
		No answer (*n* = 41)	2.4	0.0	0.0	7.3	87.8

* The results in the rows do not add up to 100%, because the identified clusters are inseparable, which means that a person from one cluster can also belong to other clusters.

**Table A3:** Cluster membership by selected characteristics of respondents (% in a cluster) (*n* = 2764).

Main Category of Independent Variables	Detailed Category	Response Category	Clusters of Risky Sexual Experiences				
			A *n* = 663	B *n* = 134	C *n* = 87	D *n* = 493	Not Belonging to Any Cluster *n* = 1667
Socio-demographic factors	Gender	Woman (*n* = 1651)	51.7	33.6	26.4	38.5	64.9
		Man (*n* = 1113)	48.3	66.4	73.6	61.5	35.1
	Age	Up to 20 years old (*n* = 616)	18.7	12.7	14.9	15.8	23.6
		21–25 years old (*n* = 1566)	62.6	64.2	51.7	57.2	54.8
		26 years old and more (*n* = 258)	10.7	9.7	17.2	12.0	8.8
		No answer (*n* = 324)	8.0	13.4	16.1	15.0	12.8
	Place of growing up	Countryside (*n* = 1502)	47.7	36.6	42.5	42.2	58.7
		A town with 20,000 inhabitants or less (*n* = 528)	21.1	21.6	19.5	24.9	17.7
		A town with 20,000–100,000 inhabitants (*n* = 459)	19.0	21.6	21.8	20.1	14.7
		A town with more than 100,000 inhabitants (*n* = 257)	11.8	19.4	14.9	12.0	8.3
		No answer (*n* = 18)	0.5	0.7	1.1	0.8	0.7
	Parents’ relationship model	Married parents who permanently lived together (*n* = 2178)	75.0	59.7	64.4	69.6	81.2
		Married parents who did not live together for months (*n* = 218)	11.3	15.7	13.8	13.0	6.5
		Unmarried parents who permanently lived together (*n* = 41)	1.2	5.2	3.4	2.6	1.1
		Unmarried parents who never lived together (*n* = 53)	2.4	6.0	6.9	4.1	1.3
		Divorced or separated parents (*n* = 169)	6.6	11.2	10.3	6.1	6.1
		One or both parents dead (*n* = 66)	2.4	1.5	1.1	3.0	2.3
		Another situation (*n* = 11)	0.5	0.7	0.0	0.4	0.3
		No answer (*n* = 28)	0.6	0.0	0.0	1.2	1.1
	Sexual orientation	Only heterosexuals *n* = 2468)	84.3	70.9	73.6	81.9	90.8
		Heterosexuals with homosexual experiences (*n* = 71)	6.3	10.4	3.4	6.1	1.1
		Bisexuals (*n* = 67)	4.5	9.7	10.3	5.5	1.6
		Homosexuals with heterosexual experiences (*n* = 13)	0.9	3.0	3.4	1.2	0.4
		Only homosexuals (*n* = 35)	2.0	4.5	8.0	3.0	0.7
		Asexuals (*n* = 44)	0.9	0.7	0.0	0.2	2.3
		No answer (*n* = 66)	1.1	0.7	1.1	2.0	3.1
	Religious commitment	Deeply religious persons who practice regularly (*n* = 313)	5.7	6.7	11.5	4.7	14.8
		Religious persons who practice regularly (*n* = 920)	24.7	12.7	24.1	20.9	38.3
		Religious persons who practice irregularly (*n* = 790)	34.1	33.6	26.4	35.1	25.2
		Religious persons who do not practice at all (*n* = 324)	15.5	18.7	13.8	15.6	9.8
		Non-believers who practice (*n* = 60)	2.1	3.7	5.7	3.4	1.7
		Non-believers who do not practice (*n* = 177)	9.8	14.9	13.8	12.0	4.4
		Hard to say (*n* = 150)	7.4	9.0	3.4	6.7	4.6
		No answer (*n* = 30)	0.6	0.7	1.1	1.6	1.2
Self-independence factors	Period of living outside the family home	Still living with parents or legal guardians (*n* = 1035)	35.4	40.3	32.2	37.9	38.5
		Less than a year (*n* = 496)	15.7	17.2	9.2	16.0	18.5
		1–2 years (*n* = 440)	15.5	17.2	20.7	17.2	15.5
		3–4 years (*n* = 496)	20.2	11.2	17.2	14.8	17.9
		5 years or more (*n* = 272)	12.7	14.2	19.5	13.4	8.5
		No answer (*n* = 25)	0.5	0.0	1.1	0.6	1.1
	Student’s support sources while studying	Only family (*n* = 1431)	48.3	47.8	46.0	45.2	54.6
		Family and persons outside the family (*n* = 90)	4.5	6.7	8.0	4.7	2.3
		student-themselves and family and (or) persons outside the family (*n* = 384)	15.4	11.2	11.5	12.0	13.2
		Only student-themselves (*n* = 825)	31.1	34.3	34.5	36.7	28.6
		No answer (*n* = 34)	0.8	0.0	0.0	1.4	1.3
	Living in a stable partner relationship or marriage	Yes (*n* = 1457)	64.3	49.3	50.6	56.2	44.9
		No (*n* = 1235)	33.8	49.3	44.8	40.6	52.3
		No answer (*n* = 72)	2.0	1.5	4.6	3.2	2.8
Ways of fulfilling the role of a student	Studied field of science	Humanities (*n* = 187)	7.8	9.0	10.3	7.1	6.3
		Engineering and technical sciences (*n* = 663)	23.2	20.1	25.3	26.0	24.3
		Medical and health sciences (*n* = 510)	18.9	21.6	17.2	19.5	18.1
		Agricultural science (*n* = 128)	3.0	1.5	2.3	6.9	4.6
		Social science (*n* = 1087)	42.7	40.3	37.9	33.9	39.7
		Exact and natural sciences (*n* = 132)	3.5	5.2	3.4	4.3	5.0
		Arts (*n* = 57)	0.9	2.2	3.4	2.4	2.0
	Average of the grades received in the previous term	Less than 3.5 (*n* = 80)	2.4	6.7	4.6	5.9	2.3
		3.5–3.9 (*n* = 657)	27.5	32.8	28.7	32.3	21.2
		4.0–4.4 (*n* = 1300)	49.2	46.3	49.4	42.6	47.3
		4.5 and more (*n* = 666)	19.6	13.4	17.2	18.1	26.4
		No answer (*n* = 61)	1.4	0.7	0.0	1.2	2.7
Attending social meetings/parties while intoxicated with psychoactive drugs	Attending social meetings/parties under the influence of alcohol	No (*n* = 1287)	22.8	22.4	29.9	32.9	56.4
		Yes, once or twice (*n* = 698)	27.1	28.4	27.6	27.2	23.8
		Yes, 3 times or more (*n* = 742)	49.9	48.5	41.4	39.4	17.8
		No answer (*n* = 37)	0.2	0.7	1.1	0.6	2.0
	Attending social meetings/parties while intoxicated with drugs	No (*n* = 2172)	60.8	17.9	47.1	58.4	86.7
		Yes, once or twice (*n* = 277)	16.1	27.6	23.0	17.4	6.7
		Yes, 3 times or more (*n* = 274)	22.9	54.5	29.9	23.5	4.5
		No answer (*n* = 41)	0.2	0.0	0.0	0.6	2.2
